# Electrophysiological Sequelae of Hemispherotomy in Ipsilateral Human Cortex

**DOI:** 10.3389/fnhum.2017.00149

**Published:** 2017-03-30

**Authors:** Ammar H. Hawasli, Ravi Chacko, Nicholas P. Szrama, David T. Bundy, Mrinal Pahwa, Chester K. Yarbrough, Brian J. Dlouhy, David D. Limbrick, Dennis L. Barbour, Matthew D. Smyth, Eric C. Leuthardt

**Affiliations:** ^1^Department of Neurological Surgery, Washington University School of MedicineSaint Louis, MO, USA; ^2^Department of Biomedical Engineering, Washington University School of MedicineSaint Louis, MO, USA; ^3^Department of Neurosurgery, University of Iowa Hospitals and ClinicsIowa City, IA, USA

**Keywords:** electrocorticography, oscillations, hemispherotomy, epilepsy, cortical physiology

## Abstract

**Objectives:** Hemispheric disconnection has been used as a treatment of medically refractory epilepsy and evolved from anatomic hemispherectomy to functional hemispherectomies to hemispherotomies. The hemispherotomy procedure involves disconnection of an entire hemisphere with limited tissue resection and is reserved for medically-refractory epilepsy due to diffuse hemispheric disease. Although it is thought to be effective by preventing seizures from spreading to the contralateral hemisphere, the electrophysiological effects of a hemispherotomy on the ipsilateral hemisphere remain poorly defined. The objective of this study was to evaluate the effects of hemispherotomy on the electrophysiologic dynamics in peri-stroke and dysplastic cortex.

**Methods:** Intraoperative electrocorticography (ECoG) was recorded from ipsilateral cortex in 5 human subjects with refractory epilepsy before and after hemispherotomy. Power spectral density, mutual information, and phase-amplitude coupling were measured from the ECoG signals.

**Results:** Epilepsy was a result of remote perinatal stroke in three of the subjects. In two of the subjects, seizures were a consequence of dysplastic tissue: one with hemimegalencephaly and the second with Rasmussen's encephalitis. Hemispherotomy reduced broad-band power spectral density in peri-stroke cortex. Meanwhile, hemispherotomy increased power in the low and high frequency bands for dysplastic cortex. Functional connectivity was increased in lower frequency bands in peri-stroke tissue but not affected in dysplastic tissue after hemispherotomy. Finally, hemispherotomy reduced band-specific phase-amplitude coupling in peristroke cortex but not dysplastic cortex.

**Significance:** Disconnecting deep subcortical connections to peri-stroke cortex via a hemispherotomy attenuates power of oscillations and impairs the transfer of information from large-scale distributed brain networks to the local cortex. Hence, hemispherotomy reduces heterogeneity between neighboring cortex while impairing phase-amplitude coupling. In contrast, dysfunctional networks in dysplastic cortex lack the normal connectivity with distant networks. Therefore hemispherotomy does not produce the same effects.

## Introduction

Since its first description in 1938, hemispheric disconnection has been used as a treatment of medically refractory epilepsy (McKenzie, 1938). This procedure has evolved from functional hemispherectomies (Rasmussen, 1983; Villemure and Rasmussen, 1993; Villemure et al., 2003) to functional hemispherotomies (Schramm et al., 1995; Villemure and Mascott, 1995; Carson et al., 1996; Kestle et al., 2000; Daniel et al., 2001; Limbrick et al., 2009). The hemispherotomy is traditionally reserved for medically-refractory epilepsy due to diffuse hemispheric disease and is thought to be effective because it prevents seizures from spreading to the contralateral hemisphere (Villemure and Rasmussen, 1993; Freeman et al., 1996; Daniel et al., 2001; Limbrick et al., 2009; Thomas et al., 2012). Hemispherotomy involves disconnection of an entire hemisphere with limited tissue resection (Limbrick et al., 2009). The majority of frontal, parietal, occipital, and temporal lobes remain *in situ* but are physically disconnected from the rest of the brain. A hemispherotomy prevents seizures from propagating and producing clinical symptoms and mitigates (but not eliminates) some complications associated with traditional hemispherectomies such as hydrocephalus (Villemure and Mascott, 1995; Limbrick et al., 2009).

Preoperative scalp electroencelphalograms (EEGs) and magnetic resonance imaging have been used to assess the epileptic cortex for decades. This has been augmented with intracranial recordings to localize seizure foci. Preoperative EEGs and intracranial recordings are typically been analyzed visually by clinical epileptologists. The use of higher level signal processing methods to assess for cortical health has not fully translated into clinical practice but is a powerful tool with great potential to further our understanding of neurophysiology and disease. Signal processing methods include basic spectral analysis to evaluate for power of cortical oscillations at various frequencies. Such methods may be used to predict seizure networks in epileptic brains (Bandt et al., 2014). Functional connectivity measures of electrical signals can be used to study short- and long-range networks in the brain. Functional connectivity measures may include cross-correlation of spontaneous signals, event-related signal correlations, and lag analysis. Evaluating how phase of one frequency affects amplitude of another is another method for network analysis. Mutual information theories and other mathematical methods can also be applied to predict networks. Functional connectivity measures can be used in EEGs and ECOG and are frequently used for functional magnetic resonance imaging studies.

The electrophysiological sequelae of traditional hemispherectomies have been evaluated in postoperative EEGs. These studies have generally shown large decreases in broadband power (Marshall and Walker, 1950; Cobb and Sears, 1960). Postoperative EEGs after hemispherotomy have shown that functionally-isolated cortex may produce epileptiform discharges but these do not propagate to the contralateral hemisphere or produce clinical effects (Marshall and Walker, 1950; Cobb and Sears, 1960). Despite its use in clinical medicine, the electrophysiological effects of hemispherotomy on ipsilateral cortical oscillations remains unclear.

To evaluate the acute electrophysiological sequelae of hemispherotomy on cortical oscillations and interrogate long-range connections to cortex, we performed intraoperative electrocorticography (ECoG) before and after hemispherotomy in two cohorts of children with epilepsy. Group 1 included children with a remote history of a perinatal infarct. For this group, we recorded from cortex on the ipsilateral hemisphere that was outside of the stroke region: i.e., peri-stroke cortex. Group 2 included children with epilepsy due to dysplastic tissue from hemimegalencephaly or Rasmussen's encephalitis. For this group, we recorded from cortex on the ipsilateral hemisphere grossly affected by the disease: i.e., dysplastic cortex. We had hypothesized that hemispherotomy would acutely (1) reduce broad-band power, (2) increase functional connectivity between adjacent electrodes and (3) disrupt cross-frequency phase-amplitude coupling within the ipsilateral disconnected cortex. The data however demonstrated that disconnection affects peri-stroke and dysplastic cortices and peri-stroke differently.

## Materials and methods

### Subjects and ethics

Five patients undergoing surgical treatment for intractable epilepsy participated in this study. Independent approval by the Washington University Institutional Review Board and the Washington University Department of Neurosurgery Ethics committee were obtained and informed consent was given for each case. Each child suffered from medically-refractory epilepsy and a hemispherotomy was recommended by an independent epilepsy committee composed of St. Louis Children's Hospital epilepsy specialists. The patient-tailored clinical operative strategy was created prior to and independently of experimental plans and the research study had no bearing or impact on the subjects' clinical care or well-being. Magnetic resonance imaging (MRI) and clinical data were collected for each subject.

Medical history and procedural information are provided in Table 1. Interpretations of traditional EEGs are provided in Supplementary Table 1. Subjects 1, 3, and 5 had a history of perinatal strokes: two had middle cerebral artery infarcts while one had an intracranial hemorrhage (Table 1, Supplementary Figure 1). Subject 2 suffered from lissencephalic hemimegalencephaly, and subject 4 suffered from Rasmussen encephalitis (Table 1, Supplementary Figure 1). Average recording duration for the procedure was 489 min and the average time between pre-FH and post-FH recordings was 273 min (Table 1).

**Table 1:** **Subjects and Procedure**.

**Subject**	**Age at surgery (y)**	**Sex**	**Seizure Onset (y)**	**Etiology**	**Antiepileptic Medications**	**Side of Procedure**	**Recording Site**	**Anesthetic**	**Surgery Time (min)**	**Minutes Between Recordings**
1	16.4	Female	8	Perinatal Right MCA Ischemic Stroke	Levetiracetam, Zonisamide, Clobazam, Lorazepam	Right	ITG	Desflurane	472	200
2	9	Female	Birth	Right Lissencephalic Hemimegalencephaly	Lamotrigine, Rufinamide, Clobazam, Clonazepam	Right	ITG	Sevoflurane	629	349
3	2.2	Female	1	Perinatal Right MCA Ischemic Stroke	Levetiracetam, Clobazam	Right	MFG	Sevoflurane	413	320
4	10.3	Female	8	Rasmussen encephalitis	Oxcarbazepine, Clobazam, Phenobarbital	Left	MFG	Sevoflurane	598	300
5	2.2	Female	1	Perinatal Right Intracranial Hemorrhage	Levetiracetam, Vigabatrin, Clonazepam, Topiramate	Right	ITG	Sevoflurane	335	195

*ITG, Inferior Temporal Gyrus; MCA, middle cerebral artery; MFG, Middle Frontal Gyrus*.

**Figure 1:**
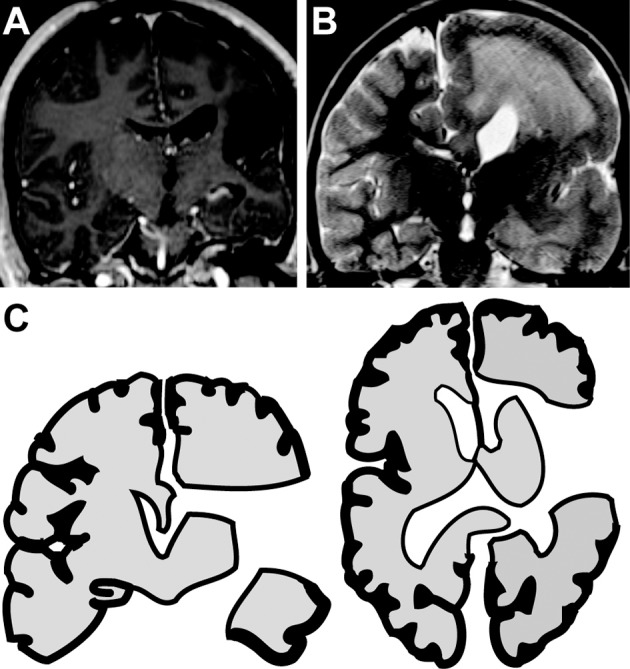
**Hemispherotomies in epileptic children. (A)** Coronal MRI reveals encephalomalacia of the right inferior frontal gyrus, superior temporal gyrus and insula in a child with a remote history of a perinatal infarct. **(B)** Coronal MRI reveals hemimegaencephaly of the right hemisphere in a child with chronic seizures of right-sided origin. **(C)** Illustration shows disconnection achieved with a hemispherotomy. The right hemisphere is disconnected from deep structures (e.g., thalamus and basal ganglia) and from the contralateral hemisphere. Hemispherotomy also includes resection of the frontal operculum, temporal operculum, amygdala and hippocampus. ECoG was recorded from tissue to be disconnected by remain *in situ*.

Subjects were divided into two cohorts based on their etiology of epilepsy and suspected nature of the recording sites. Group 1 included three subjects who had suffered remote perinatal cerebral infarcts and subsequent encephalomalacia of the infarcted gyrus (Figure 1A). For these subjects, ECoG was recorded in a remaining gyrus that was not visibly damaged by the remote stroke. Hence, recordings were performed on cortex that radiographically and visually appeared intact. The second cohort included two subjects with epilepsy syndromes and visibly dysplastic cortex. For these two subjects, all areas available for recording were dysplastic. One child had hemimegalencephaly (Figure 1B) and a second had Rasmussen's encephalitis. For these subjects, ECoG was recorded from dysplastic cortex.

### Equipment

ECoG signals were recorded and digitized from a pair of implanted 4-contact electrode array at 2,400 samples per second, using the G.USB biosignal amplifier (G-TEC, Austria). Unfiltered data were stored on a custom computer running BCI2000 software (Schalk laboratory, USA). The ECoG electrodes (PMT® Corporation) were used to acquire ECoG signals. Electrodes were made of platinum, each 4 mm in diameter with 2.3 mm exposed to the cortical surface and spaced apart by 1 cm.

### Craniotomy and electrode placement

Each subject underwent a craniotomy under general anesthesia. Sevoflurane was used for 4 subjects and desflurane was used for 1 subject. Gas concentration monitors allowed for stable anesthesia. Prior research has demonstrated that dynamic physiology in cortical and thalamocortical circuitry is maintained despite general anesthesia (Breshears et al., 2010). Standard neurosurgical and neuroanesthesia practices were maintained to ensure similar craniotomy conditions. After removal of the cranial window and dural incision, the selected gyrus was exposed, a 4-contact ECoG electrode strip was placed on a selected gyrus that would be disconnected but not resected in the course of the hemispherotomy. ECoG was recorded from the inferior temporal gyrus in 3 subjects and the middle frontal gyrus in 2 subjects. Photographs, intraoperative anatomical landmarks and MRI neuro-navigation coordinates ensured that post-disconnection recordings were performed in the same location. An additional 4-contact distant electrode was inverted and secured onto the cranial dura to serve as a reference/control.

### Intraoperative *in vivo* ECoG

Intraoperative *in vivo* ECoG data were collected from the cortex under general anesthesia before and after hemispherotomy. Two distant intracranial dural electrodes were selected as ground and reference contacts and subject's left lower extremity was grounded. Surgical manipulation and operating room activity were halted during recordings. Care was taken to ensure sufficient contact between the recording sites and electrode surfaces. Adequate signal voltage recordings and signal-to-noise ratios were confirmed by an online BCI2000 graphics user interface. For each recording, 5 min epochs were collected with minimal operating room activity. After the disconnection, the ECoG electrodes were replaced onto the same locations they were in during the pre-hemispherotomy recording. Physical landmarks such as vessels and sulci, photographs and neuronavigation stereotactic coordinates were available to ensure the electrodes were positioned in the appropriate location.

### Hemispherotomy

The hemispherotomy began with opening of the Sylvian fissure and resection of the frontal and temporal opercula. The lateral ventricle was accessed and opened circumferentially from the anterior part of the temporal horn to the anterior portion of the frontal horn. This was followed by a selective amygdalohippocampectomy. After locating the pericallosal arteries, a complete parasagittal transventricular corpus callosotomy was performed, tracking the anterior cerebral arteries proximally to disconnect the frontal basal tissue as well. When present, the insula was decorticated. Completion of a hemispherotomy ensured cortical disconnection from deep gray-matter structures, limbic system, and the contralateral hemisphere (Figure 1C). Hemispherotomies were performed using neuro-navigation guidance, surgical microscope, and expanded-field surgical telescopes. Hemispherotomies were performed by a board-certified pediatric neurological surgeon. Postoperative MRIs confirmed complete disconnection in all subjects.

### Biomedical signal processing

All signal processing scripts were custom written in MATLAB, unless otherwise noted. Signals from every electrode were visually inspected and those identified as having predominately poor signal-to-noise characteristics (amplitude greater than 10x that of the majority of electrodes in the array) were excluded from further analysis. Data were also excluded if there were intraoperative inconsistencies or incomplete data. The signal at each of the four cortical electrodes was re-referenced to the common mean of the cortical electrode to minimize common sources of noise from the signals. All re-referenced ECoG voltages where then processed through digital 0.25 Hz high-pass, 500 Hz low-pass, and 60-Hz-notch band-stop digital butterworth-filters. Traditional frequency bands were defined as δ (0.5–4 Hz), θ (4–7 Hz), α (8–12 Hz), β (12–30 Hz), γ (30–96 Hz), mid-γ (65–75 Hz) and high-γ (83–93 Hz). In addition to comparing power spectral densities (PSDs) before and after hemispherotomy, PSD was also compared between peri-stroke cortex (group 1) and dysplastic cortex (group 2).

Spectral analysis was done using the Welch's power spectrum density estimate method (Welch, 1967). After power spectral densities of rereferenced signal voltages were calculated, logarithmic PSD was normalized to pre-hemispherotomy condition at 0.44 Hz and plotted.

Mutual information is a quantification of the information gained about a random variable X from measurement of a second variable Y (Cover and Thomas, 1991; Leuthardt et al., 2009). Mutual information was used to assess functional connectivity between adjacent signals before and after hemispherotomy. An MI of zero indicates that two signals are completely independent variables while the MI between X and Y will be > 0 if knowing Y reduces the uncertainty of X. Information from measurements are represented as entropy (H), which can be used to calculate the MI between X and Y:

$$\begin{array}{l} \text{H}\left(\text{X}\right)=\underset{-\infty }{\int^{\infty }}{\text{P}}_{x}(\text{x})\;\text{ log}2\left({\text{P}}_{x}\left(\text{x}\right)\right)\\ \text{ MI}\left(\text{X},\text{Y}\right)=\text{H}\left(\text{X}\right)+\text{H}(\text{X}/\text{Y}) \end{array}$$

where H(X/Y) is the information of X gained by measurement of Y and P_X_(x) is the probability that X = x in system X. Mutual information was calculated between the voltage signals.

To eliminate potential spurious artifact caused by volume conduction of simultaneous voltage fluctuations, MI was also calculated after orthogonalization of the signals (Hipp et al., 2012). The signal components that shared the same phase were removed for each pair of signals to generate orthogonalized signals. For signals X and Y, the orthogonalized signal of Y, Y_⊥_(t,f), is the difference between Y(t,f) and the part of Y(t,f) that points into the direction of X(t,f). This is calculated:

$$\begin{array}{l} {\text{Y}}_{⊥X}\left(t,f\right)=imag\left(Y\left(t,f\right)\frac{X{\left(t,f\right)}^{\text{*}}}{\left|X\left(t,f\right)\right|}\right)\underset{˙}{\hat{e}}⊥X\left(t,f\right)\\ \;\underset{˙}{\hat{e}}⊥X\left(t,f\right)=\frac{iX\left(t,f\right)}{\left|X\left(t,f\right)\right|} \end{array}$$

The ^*^ indicates the complex conjugate, *imag* is the imaginary part of a complex number, and ê⊥X(t,f) is the complex number pointing orthogonal the direction of X in a clockwise direction (Hipp et al., 2012). To calculate MI index, mutual information for each electrode was re-referenced to 1 Hz value at pre-hemispherotomy condition and plotted.

Phase-amplitude coupling signal analyses were performed to evaluate how low frequency oscillations phases modulate high frequency oscillation amplitudes, as previously described (Canolty et al., 2006; Tort et al., 2010; Daitch et al., 2013; Hawasli et al., 2015). Phase-amplitude coupling was assessed for every frequency pair in a 2-dimensional frequency space. Spectral decomposition of re-referenced signals was performed using Gabor wavelet filtering which produced instantaneous amplitude and phase estimates at time points for each frequency (Canolty et al., 2006). Modulation index was calculated by applying an entropy measurement to determine divergence of the observed amplitude distribution from the uniform distribution (Tort et al., 2010). Magnitude of phase-amplitude coupling was represented as a modulation index Z-score, which was the dependence of amplitude of one variable on the phase of another variable. Data are shown as modulation index Z-score or change in modulation index Z-score from pre-hemispherotomy condition (Canolty et al., 2006). Modulation index Z-score of 3.9 or greater indicated significant phase-amplitude coupling (*p* = 0.0494, *Z* = 3.9) after correcting for multiple comparisons.

### Statistics

To assess for statistical differences between power and MI data, multi-way analysis of variance tests (ANOVA) were performed. When significant interactions or main effects were measured, planned *post hoc* Tukey's honestly significant difference tests were performed. All significant differences found on *post hoc* comparisons had *p* < 0.05. Modulation index Z-score of 3.9 or greater indicated significant phase-amplitude coupling (*p* = 0.0494) after correcting for multiple comparisons. Differences in modulation index Z-scores before and after hemispherotomy were assessed by Kruskal-Wallis one-way analysis of variance. *P*-values, after false-discovery rate multiple comparison correction, degrees of freedom and statistics are reported in the manuscript. Statistical values with *p* < 0.05 indicated statistical significance.

## Results

### Peri-stroke and dysplastic cortices display different baseline power spectra

**Figure 2:**
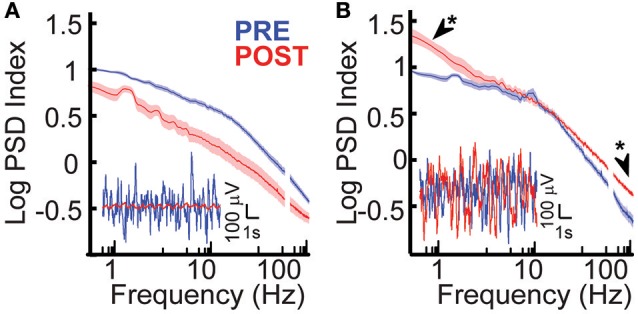
**Effects of hemispherotomy on broad-band power in epileptic children. (A)** Hemispherotomy reduces broad band power of oscillations in peri-stroke cortex-group 1 [*F*_(1, 29678)_ = 1.51 × 10^4^, *p* < 10^−30^]. Inset traces show exemplar broad-band cortical oscillations that were reduced in power by hemispherotomy in peri-stroke cortex. **(B)** Hemispherotomy significantly affected frequency-dependent PSD in subjects with dysplastic cortex [*F*_(1348, 18886)_ = 2.96, *p* < 2.5 × 10^−224^]. Exemplar traces show that cortical oscillations are not reduced after hemispherotomy in dysplastic tissue. Data represent means ± standard error. Asterisks indicates low-δ and high-γ band power were greater after hemispherotomy by *post hoc* analysis (*p* < 0.05).

Baseline electrophysiological properties of peri-stroke cortex and dysplastic cortex were compared. When all cohorts and conditions were compared, group 1 (peri-stroke cortical ECoG) and group 2 (dysplastic cortical ECoG) showed significant baseline electrophysiological differences in power. At baseline, the scale-free power spectral density curves of groups 1 and 2 had different appearances (Figures 2A,B, *blue*) and there was a frequency-dependent difference in PSD between groups 1 and 2 [*ANOVA, F*_(1348, 48564)_ = 1.83, *p* = 2.83 × 10^−65^]. *Post-hoc* analysis showed that prior to hemispherotomy, PSD of group 1 was significantly greater than the PSD of group 2 across the frequency spectra (*p* < 0.05).

### Hemispherotomy differentially influences broad spectrum power in peri-stroke and dysplastic cortices

The role of long-range inputs into cortex and the effects of hemispherotomy on cortical physiology were evaluated in epileptic children by recording ipsilateral ECoG before and after disconnection. Hemispherotomy led to a significant overall decrease in PSD in group 1, the peri-stroke group, relative to baseline [*F*_(1, 29678)_ = 1.51 × 10^4^, *p* < 10^−30^; Figure 2A]. These deceases in power were observed across the frequency spectra. There was also a significant frequency-dependent effect of hemispherotomy on PSD within the dysplastic group [*F*_(1348, 18886)_ = 2.96, *p* < 2.5 × 10^−224^]. However, this effect was different from the group 1 as demonstrated in Figure 2B. *Post-hoc* analysis showed that PSD was greater *after* hemispherotomy in low-δ and high-γ frequencies (Figure 2B). Hence, hemispherotomy reduced broad-band PSD in peri-stroke cortical tissue suggesting that long-range inputs into peri-stroke cortex facilitates broadband PSD. In contrast, for dysplastic cortex, disconnection increased PSD in low and high frequency bands but had no effect on frequencies in between. This suggests long-range inputs impacts power of oscillations in a frequency- and disease-specific manner.

### Peri-stroke cortex displays less baseline local functional connectivity than dysplastic cortex

Functional connectivity measures are statistical methods that can be used to estimate interactions between neighboring regions of cortex and have been applied to electrophysiology and functional neuroimaging studies (Cover and Thomas, 1991; Ortega et al., 2008; Friston, 2011; Greenblatt et al., 2012). To assess functional connectivity, mutual information (MI) shared with adjacent regions of cortex was measured before and after hemispherotomy. MI between adjacent electrodes were measured before and after hemispherotomy to assess for the role of long-range inputs on spatial heterogeneity in cortical signals. Baseline local electrophysiological functional connectivity of peri-stroke cortex and dysplastic cortex were compared. When all cohorts and conditions were compared, group 1 (peri-stroke cortical ECoG) and group 2 (dysplastic cortical ECoG) showed significant baseline electrophysiological differences in functional connectivity. There was a pathology- and condition (pre- vs. post-hemispherotomy) –dependent difference in functional connectivity between groups 1 and 2 [*ANOVA, F*_(1, 1300)_ = 12.6, *p* = 3.9 × 10^−4^]. *Post-hoc* analysis showed MI prior to hemispherotomy of group 1 was significantly less than MI of group 2 across the frequency spectra (*p* < 0.05). These findings show that local functional connectivity is greater in dysplastic cortex than peri-stroke cortex.

### Hemispherotomy leads to increased local functional connectivity in peri-stroke cortex in low frequencies

**Figure 3:**
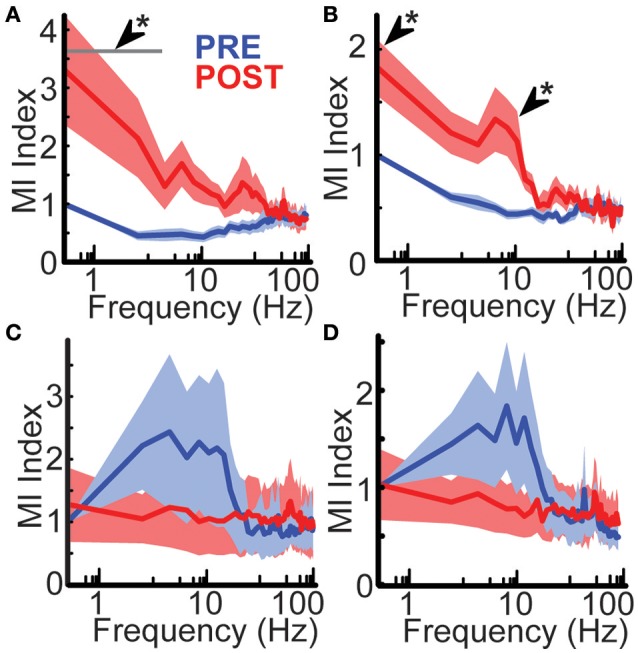
**Hemispherotomy increased low-frequency functional connectivity in peri-stroke cortex. (A)** Hemispherotomy increased δ-band functional connectivity in peri-stroke cortex. Hemispherotomy had a significant frequency-dependent effect on mutual information shared with signal from neighboring cortex in peri-stroke tissue [*F*_(49, 800)_ = 2.13, *p* < 10^−4^]. **(B)** Hemispherotomy increased low-frequency connectivity in peri-stroke cortex when measuring mutual information of orthogonalized signal. Hemispherotomy had a significant main effect on orthogonalized mutual information shared with signal from neighboring cortex in peri-stroke tissue [*F*_(49, 800)_ = 2.81, *p* < 10^−8^]. Hemispherotomy had no significant effects on mutual information of **(C)** raw signal or **(D)** orthogonalized signal in dysplastic cortex. MI Index represents mutual information shared with adjacent cortex normalized to 0.5 Hz frequency at pre-hemispherotomy condition. Data represent means ± standard error. ^*^P < 0.05, *post hoc* Tukey's HSD test, post- vs. pre-conditions.

To assess the effects of hemispherotomy on local functional connectivity, MI was measured before and after disconnection. Hemispherotomy of peri-stroke cortex (group 1) led to a frequency-dependent change in MI shared with adjacent cortex when compared with baseline [*F*_(49, 800)_ = 2.13, *p* = 1.86 × 10^−5^; Figure 3A]. *Post-hoc* analysis confirmed significantly increased MI within the δ frequency-band after disconnection. To reduce potential spurious artifacts caused by volume conduction of simultaneous voltage fluctuations, MI was also calculated after orthogonalization of the signals (Hipp et al., 2012).

Similarly, hemispherotomy led to a frequency-dependent difference in MI of orthogonalized signal shared with adjacent cortex [*F*_(49, 800)_ = 2.81, *p* = 2.43 × 10^−9^, Figure 3B]; *post-hoc* analysis confirmed significantly increased MI of orthogonalized signal within the δ and Θ frequency-bands. These findings suggest functional disconnection of the hemisphere in peri-storke cortex leads to ipsilateral loss of low-frequency inputs that normally facilitate topographic heterogeneity and autonomy of oscillatory behavior in mesoscale regions of cortex.

### Hemispherotomy does not alter ipsilateral functional connectivity in dysplastic cortex

To assess the role of long-range connections in functional connectivity for dysplastic cortex, MI was measured before and after hemispherotomy. hemispherotomy had no significant effect on frequency-dependent MI [*F*_(49, 500)_ = 0.35, *p* = 1] nor a main effect of condition on MI in dysplastic cortex [*F*_(1, 500)_ = 0.11, *p* = 0.73; Figure 3C]. Evaluation of orthogonalized signal also revealed no effect of hemispherotomy on frequency-dependent MI [*F*_(49, 500)_ = 0.61, *p* = 0.98; Figure 2D] nor a main effect on MI [*F*_(1, 500)_ = 1.23, *p* = 0.27]. Hence, hemispherotomy had no significant effects on functional connectivity in dysplastic cortex. This suggests that dysplastic cortex lacks the low-frequency inputs which would normally facilitate topographic heterogeneity in human cortex.

### Hemispherotomy reduces cross-frequency PAC in ipsilateral peri-stroke cortex

**Figure 4:**
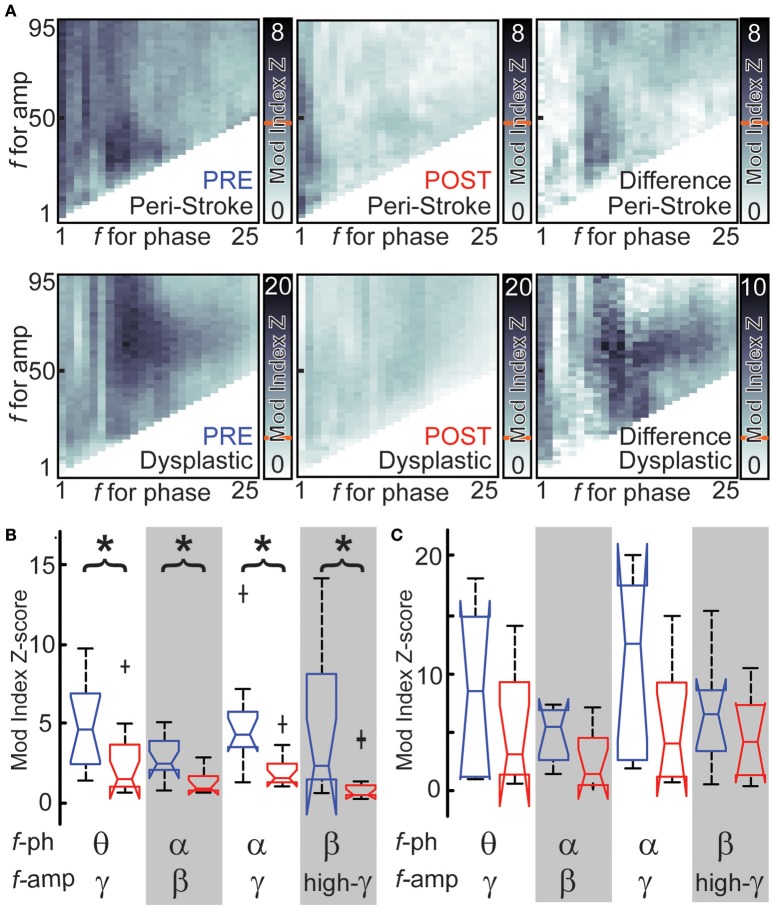
**Hemispherotomy reduces cross-frequency PAC in peri-stroke cortex. (A)** Cross-frequency phase- amplitude exemplar data are shown for peri-stroke (*top*) and dysplastic cortex (*bottom*) before (*blue*) and after (*red*) hemispherotomy. The difference in cross-frequency PAC before and after is also shown (*right*). Shading represents modulation index or change at frequencies for phase and frequencies of amplitude. Red line indicates Mod index Z-score necessary for significant PAC. **(B)** Hemispherotomy reduces cross-frequency PAC between several canonical frequency bands in peri-stroke tissue **(C)** but not dysplastic tissue **(D)**. Box plot data represent median ± maximum/minimum and quartiles. ^*^P < 0.05, post- vs. pre-conditions, Kruskal-Wallis one-way analysis of variance, multiple comparison-corrected.

Cross-frequency phase-amplitude coupling (PAC) between low-frequency phases and higher frequency amplitude may serve as a mechanism to transfer information from broad-scale brain networks to local cortical circuits (Canolty and Knight, 2010). PAC with γ amplitude has been implicated in neuronal firing, cortical activation and task completion (Canolty et al., 2006; Cardin et al., 2009; Breshears et al., 2010). If broad-scale networks modulate cortical PAC, we hypothesized that hemispherotomy should reduce PAC. PAC for baseline peri-stroke tissue was largest for δ phase-γ amplitudes (median [med] modulation index z-score = 4.29, inter-quartile range [IQR] = 6.9) and for θ phase-γ amplitude (med = 4.4, IQR = 4.5; Figure 4A, *top*). In peri-stroke cortex, hemispherotomy significantly reduced cross-frequency PAC (Figure 4A, *top*). Hemispherotomy reduced PAC between θ phase and γ amplitude [$\chi_{\left(1\right)}^{2}$ = 6.5; *p* = 0.03], α phase and β amplitude [$\chi_{\left(1\right)}^{2}$ = 9.0; *p* = 0.01], α phase and γ amplitude [$\chi_{\left(1\right)}^{2}$ = 10.1; *p* = 0.01], and β phase and high-γ amplitude [$\chi_{\left(1\right)}^{2}$ = 7.4; *p* = 0.02; Figure 4B]. Hence, hemispherotomy reduced PAC in peristroke tissue suggesting that broad-scale networks modulate cortical PAC in peri-stroke tissue.

Hemispherotomy in dysplastic cortex (group 2) showed also significant PAC at baseline at many frequencies. PAC was largest between θ phase and α-γ amplitudes and between α phase and γ amplitude (Figure 4A, *bottom*). Although hemispherotomy led to a qualitative reduction in PAC throughout much of the spectrum in select recordings (Figure 4A, *bottom*), the reduction in PAC was insignificant (Figure 4C) when taken as a group. Hence, hemispherotomy leads to consistent decreases cross-frequency PAC in peri-stroke tissue but not in dysplastic tissue. This suggests that large-scale networks modulate PAC in peri-stroke tissue but not in dysplastic cortex.

## Discussion

Hemispherotomy is a treatment modality reserved for select group of pediatric epilepsy patients. While it can be associated with significant morbidities (e.g., Hemiparesis, visual/sensory deficits, and speech/cognitive deficits), the procedure may reduce the frequency or entirely eliminate clinical seizures in children suffering from life-debilitating epilepsy and thus enable a more normal development. From an electrophysiological standpoint, anecdotal and prior clinical data have shown that local epileptiform activity may persist in the disconnected cortex after a hemispherotomy but these electrographic events do not propagate to the contralateral hemisphere (Cobb and Sears, 1960; Carson et al., 1996; Daniel et al., 2001; Limbrick et al., 2009). Beyond the assessment of how a hemispherotomy impacts the seizures, there has been little done to study the manner in which the cortical physiology of the pathologic hemisphere is actually altered by this significant disconnection. Here we evaluated the effects of hemispherotomy on fundamental ECoG measurements to (1) evaluate how hemispherotomy affects pathology-specific cortical physiology and (2) examine contributions of long-distance circuits on oscillations. These unique human experiments highlights that hemispherotomy has disease-specific effects on oscillatory power, functional connectivity and local signal-processing. These differential effects give clues to the manner in which central regions in the brain interact with cortex and the distinct manner in which various diseases alter those interactions.

Prior lesion studies in humans have demonstrated that there are distinct effects that white matter and gray matter connections have on the nature of cortical oscillations. There are some interesting similarities and differences between those studies and the current work. In the study performed by Hawasli et al. white matter and gray matter disconnections were performed immediately adjacent to normal cortex in a temporal lobe (a region of the brain that was necessarily going to be resected to access a deeper pathology) (Hawasli et al., 2015). Thus, while the white and gray matter lesions were local (<1 cm from recorded cortical site) and the disconnected cortex was demonstrably normal for Hawasli et al. in this experiment the disconnection is distant from the recorded cortex and the cortex is abnormal to varying degrees (peri-stroke cortex more normal, while the dysplastic cortex is quite abnormal). In the setting of peri-stroke recordings, there are a number of similarities with the cortical changes seen with white matter disconnection of normal cortex. Both studies showed a reduction in power after disconnection and an increase in connectivity. The parallels in these studies suggest that central regions, such as the thalamus, are still maintaining interactions with peristroke cortex to enhance cortical power and maintain functional diversity. In the setting of dysplastic cortex, the findings are quite distinct when compared to white and gray matter disconnections for normal cortex. When dysplastic cortex is disconnected, there are no power alteration in the alpha and beta rhythms that are most commonly associated with thalamocortical circuits (Andersen and Sears, 1964; Andersen and Andersson, 1968; Canolty et al., 2006). Also, the hemispherotomy in patients with dysplastic cortex did not alter cortical connectivity. We interpret these findings to suggest that in the setting of severe cortical derangement that the thalamocortical interactions are much more profoundly compromised prior to hemispherotomy disconnection than is the case in perinatal infarct.

Another interesting finding in this work is the effect that hemispherotomy had on phase amplitude coupling (PAC). Historically, PAC has been thought to be a form of cortico-cortical communication. It has been suggested to serve as a mechanism to transfer information from large-scale distributed brain networks to the fast, local cortical processing required for effective computation and synaptic modification (Canolty and Knight, 2010). This phenomenon has been implicated in neuronal firing, cortical activation, task completion, and alterations in consciousness (Canolty et al., 2006; Cardin et al., 2009; Breshears et al., 2010). Recent work challenges these notions (Hawasli et al., 2015) Hawasli et al. report that when a one-centimeter region of normal cortex was circumferentially disconnected from adjacent cortex, the PAC was significantly increased. If PAC were a mechanism for cortico-cortical communication, PAC should to be reduced. Similarly, we show in this work that when peri-stroke cortex (which appears to have more normal thalamocortical interactions) is globally disconnected, cortical-cortical connectivity is increased but PAC is significantly decreased. Thus, when cortico-cortical communication is augmented by a central disconnection, the PAC is suppressed. When the thalamocortical interactions are not present and cortex is substantively abnormal, as is the case with the dysplastic cortex, these relationships are not observed. Taken together, these findings support notion that modulation of PAC is associated with local cortex, rather than with long-range cortico-cortical communication.

These electrophysiologic findings potentially could be clinically informative of the functional health of the hemisphere being disconnected. As seen in Figure 3, there are clear differences at baseline in the mutual information measures between peri-stroke cortex and dysplastic cortex. Where peri-stroke cortex has lower levels of shared mutual information, there are much higher levels of shared information for the dysplastic cortex. This likely represents that the peri-stroke region has more normal thalamocortical interactions and as a result has maintained more normal functional separation of cortical regions. Dysplastic cortex has lost this thalamoocortical relationship and thus has more homogenous “noisy” cortical activity. These differences may potentially inform the functional consequences of the hemispherotomy. If the hemisphere has more physiologic structure the patient may be at higher risk for losing some cognitive function that is still operant in that region. If it appears that mutual information measures are high, then the probability that these regions are functionally relevant is lower. At this time these can only be hypotheses, and will require more detailed clinical analysis and outcomes assessment to assess their merit.

Several limitations in this study deserve attention. Despite the chronic effects of disorders such as seizures and antiepileptic medications on the human brain, cortical oscillations and amplitude modulation are observed in epilepsy patients with invasive monitoring (Pfurtscheller and Berghold, 1989; Crone et al., 1998a,b). Volume conduction presents problems functional connectivity measures between signals recorded from adjacent regions of cortex (Buzsáki et al., 2012). Despite this omnipresent confounder in electrocorticography, many lines of evidence support that the findings presented here cannot be entirely attributed to volume conduction. First, orthogonalization of the signal has been shown to eliminate the effects of volume conduction (Hipp et al., 2012). Then after removing common, instantaneous, signal among electrodes, hemispherotomy affected power differently for the two groups. An increase in power cannot be explained by and is contrary to volume-conducted effects. Frequency band-specific findings changes in functional connectivity after transections are unlikely to be explained by persistent volume conduction. Many studies have evaluated high gamma band range up to and even beyond 150 Hz. This study was limited to gamma frequencies below 95 Hz due to noise within the operating room environment. The study is further limited by its small sample size. Future studies to increase sample size through multi-center collaboration would be beneficial. Future studies using alternate connectivity measures through functional magnetic resonance imaging resting state networks are also warranted.

## Ethics statement

This study was carried out in accordance with the recommendations of the Washington University Human Research Protection Organization Institutional Review Board with written informed consent from all subjects. All subjects gave written informed consent in accordance with the Declaration of Helsinki. The protocol was approved by the Washington University Department of Neurosurgery Institutional Review Board committee and the Washington University Human Research Protection Organization Institutional Review Board.

## Author contributions

AH, DLB, MS, and EL conceived and designed the experiments. All authors contributed data collection and analysis. MS and EL supervised the research. All authors wrote the manuscript. MS and EL contributed equally.

## Funding

This study was supported by grant funding support from the National Institute of Health (5T32NS007205-32, AH; R01-DC009215, DLB) and National Science Foundation (EFRI-1137211, EL). The funders had no role in study design, data collection and analysis, decision to publish, or preparation of the manuscript. The authors declare no relevant competing financial interests.

### Conflict of interest statement

The authors declare that the research was conducted in the absence of any commercial or financial relationships that could be construed as a potential conflict of interest. The reviewer MS declared a past collaboration with some of the authors to the handling Editor, who ensured that the process met the standards of a fair and objective review.

## Supplementary material

Supplementary Figure 1 **Brain magnetic resonance imaging of epileptic children**. Coronal T2- weighted MRI subjects 1–5. R indicates right side.

Supplementary Table 1 **Effects of hemispherotomy on EEG**.

## Abbreviations

ECoG: electrocorticography
NMI: normalized mutual information
PSD: power spectral density estimate
PAC: phase-amplitude coupling. Band-specific δ, θ, α, μ, β, γ, and high-γ were defined as 0.5–4, 4–7, 7–12, 8–12, 12–30, 30–100, and 70–90 Hz, respectively.
