# Supplementary figures and images for: Electrophysiological Sequelae of Hemispherotomy in Ipsilateral Human Cortex
Source: Front Hum Neurosci. 2017 Mar 30;11:149. doi: 10.3389/fnhum.2017.00149 (PMC5371676; doi:10.3389/fnhum.2017.00149)

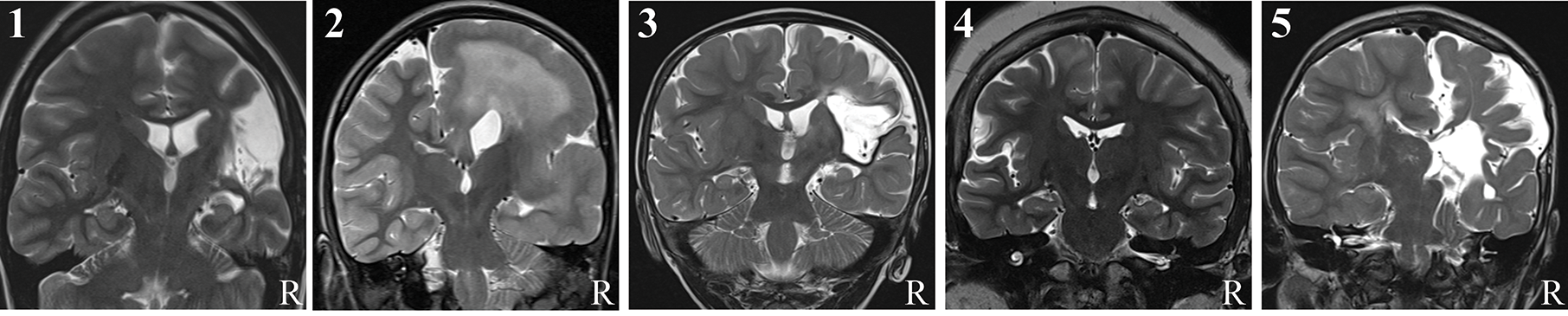

Supplement: Supplementary Figure 1 — Brain magnetic resonance imaging of epileptic children. Coronal T2- weighted MRI subjects 1–5. R indicates right side. [file Image1.TIF]
