# Supplementary material for: Electrophysiological Sequelae of Hemispherotomy in Ipsilateral Human Cortex
Source: Front Hum Neurosci. 2017 Mar 30;11:149. doi: 10.3389/fnhum.2017.00149 (PMC5371676; doi:10.3389/fnhum.2017.00149)
Supplement: Supplementary Table 1 — Effects of hemispherotomy on EEG. [file Table1.DOCX]

**Supplementary Table 1.**

| Subject | Background EEG before Hemispherotomy | Background EEG after Hemispherotomy |
| --- | --- | --- |
| 1 | Left: 20-60 microvolt theta and alpha activity.  Right: 30-60 microvolt theta activity.  Right-side onset seizures that generalized. | Left: 20-80 microvolt alpha activity.  Right: 10-30 microvolt 1-3 Hz delta activity.  No seizures observed. |
| 2 | Left: 100-150microvolt delta, theta and alpha activity.  Right: 200-400 microvolt delta and theta activity with epileptiform discharges.  Right-side onset seizures that generalized. | Left: 60-100-150 microvolt delta, theta and higher frequency activity.  Right: 50-300 microvolt delta and theta activity with epileptiform discharges.  Right-side seizures; No spread to other side. |
| 3 | Medium amplitude (Right>Left) polymorphic delta activity.  Seizure activity noted on right side. | Left : Low to medium amplitude delta, alpha and beta activity.  Right side: attenuated activity.  No seizures observed. |
| 4 | Left: 20-120 microvolt theta, alpha and beta frequencies with frequency seizure activity.  Right: Not reported.  Left-side onset seizures that generalized. | Left: 20 -40 microvolt theta activity and 40-80 microvolt delta activity.  Right20-40 microvolt alpha activity.  No seizures. |
| 5 | Left: 50-300 microvolt delta and theta activity.  Right: 50-400 microvolt delta and theta activity.  Right-side onset seizures that generalized. | Right-sided epileptiform activity noted without spread to other side and no clinical correlate. |
